# [Expression of Concern] MUC1 is a downstream target of STAT3 and regulates lung cancer cell survival and invasion

**DOI:** 10.3892/ijo.2025.5829

**Published:** 2025-12-03

**Authors:** Jingchun Gao, Matthew J. McConnell, Bin Yu, Jiannong Li, Justin M. Balko, Esther P. Black, Joseph O. Johnson, Mark C. Lloyd, Soner Altiok, Eric B. Haura

Int J Oncol 35: 337-345, 2009; DOI: 10.3892/ijo_00000345

Following the publication of the above paper, it was drawn to the Editor's attention by a concerned reader that the Bcl-2 protein bands shown for the HCC827 cell line in the western blots in Fig. 3A on p. 342 were unexpectedly similar to the Akt protein bands shown for the A549 cell line in Fig. 3B. In addition, the Bcl-2 protein bands shown for the H358 and HCC827 cell lines (the left-hand and middle gels) possibly contained a pair of mutually overlapping protein bands, and the β-actin control bands featured in the left-hand and middle lanes of Fig. 3A for the H358 and HCC827 cell lines, and the β-actin control bands featured in the middle and right-hand lanes for the H358 and HCC827 cell lines in Fig. 1C on p. 340, were similarly more similar than might have been expected.

The authors were contacted by the Editorial Office to offer an explanation for this possible anomaly in the presentation of the data in this paper, although up to this time, no response from them has been forthcoming. Owing to the fact that the Editorial Office has been made aware of potential issues surrounding the scientific integrity of this paper, we are issuing an Expression of Concern to notify readers of this potential problem while the Editorial Office continues to investigate this matter further.

